# Modeling the Relations Among Morphological Awareness Dimensions, Vocabulary Knowledge, and Reading Comprehension in Adult Basic Education Students

**DOI:** 10.3389/fpsyg.2016.00086

**Published:** 2016-02-04

**Authors:** Elizabeth L. Tighe, Christopher Schatschneider

**Affiliations:** ^1^Institute for the Science of Teaching and Learning, Arizona State UniversityTempe, AZ, USA; ^2^Florida Center for Reading Research, Florida State UniversityTallahassee, FL, USA

**Keywords:** adult basic education, morphological awareness, reading comprehension, structural equation modeling, vocabulary knowledge

## Abstract

This study extended the findings of Tighe and Schatschneider (2015) by investigating the predictive utility of separate dimensions of morphological awareness as well as vocabulary knowledge to reading comprehension in adult basic education (ABE) students. We competed two- and three-factor structural equation models of reading comprehension. A three-factor model of real word morphological awareness, pseudoword morphological awareness, and vocabulary knowledge emerged as the best fit and accounted for 79% of the reading comprehension variance. The results indicated that the constructs contributed jointly to reading comprehension; however, vocabulary knowledge was the only potentially unique predictor (*p* = 0.052), accounting for an additional 5.6% of the variance. This study demonstrates the feasibility of applying a latent variable modeling approach to examine individual differences in the reading comprehension skills of ABE students. Further, this study replicates the findings of Tighe and Schatschneider (2015) on the importance of differentiating among dimensions of morphological awareness in this population.

## Introduction

There is a relative dearth of rigorous research investigating the component reading skills of struggling adult readers. In addition to decoding skills (Mellard et al., 2010; Sabatini et al., 2010; Mellard and Fall, 2012; Fracasso et al., in press; To et al., in press), recent research has identified morphological awareness (Tighe and Binder, 2015; Fracasso et al., in press; To et al., in press) and vocabulary knowledge (Mellard et al., 2010; Mellard and Fall, 2012; Taylor et al., 2012; Hall et al., 2014; Fracasso et al., in press) as important predictors of reading comprehension in this population. However, there are several limitations to these studies: (1) only a single study has included both morphological awareness and vocabulary knowledge as predictors of reading comprehension; (2) none of the studies have utilized a latent variable modeling approach to investigate predictors of reading comprehension; and (3) all of the research has treated morphological awareness as a unidimensional construct. Tighe and Schatschneider (2015) identified two distinct latent dimensions of morphological awareness (real word morphological awareness and pseudoword morphological awareness) that were also separable from vocabulary knowledge in a sample of adult basic education (ABE) students. Thus, the current study addressed previous limitations and extended the results of Tighe and Schatschneider (2015) by including separate dimensions of morphological awareness as well as vocabulary knowledge to estimate the unique and shared contributions of these constructs to reading comprehension in a sample of ABE students.

### Adult Basic Education Programs and Student Characteristics

Adult basic education programs are designed to provide instruction to adults (ages 16 and older and not concurrently enrolled in K-12 education) in order to complete a General Educational Development certificate (GED; or high school equivalency degree). These programs cater to a heterogeneous group of learners in terms of age, race/ethnicity, learning disability status, English language learner status, educational background, and motivations for pursuing a GED certificate (Lesgold and Welch-Ross, 2012; Tighe et al., 2013). Many of these programs suffer from under-funding, over-crowding, high teacher turnover rates, high student attrition rates, and a lack of empirically based, standardized curricular materials and instructional practices (Lesgold and Welch-Ross, 2012). The heterogeneity of the learner characteristics and the paucity of rigorous research addressing the literacy skills and needs of this population present a challenge for delivering high-quality and consistent instruction within ABE programs.

### Morphological Awareness and Reading Comprehension

Recently, a few studies have investigated the component reading skills of ABE students and have identified morphological awareness, a conscious understanding of small units of meaning (e.g., prefixes, suffixes; Carlisle, 2000), as an important predictor of adults’ reading comprehension skills (Tighe and Binder, 2015; Fracasso et al., in press; To et al., in press). All of these studies utilized parallel or hierarchical regression analyses, which do not allow the researchers to model measurement error in the morphological awareness and reading comprehension assessments. Further, all of these studies relied on a single assessment of reading comprehension in the analyses even though reading comprehension is a broad construct with multiple types of assessments (i.e., narrative versus expository texts; cloze procedure versus multiple choice questions; Cutting and Scarborough, 2006). Despite these limitations, two of these studies (Tighe and Binder, 2015; To et al., in press) reported that morphological awareness contributed substantial unique variance to reading comprehension beyond other reading-related constructs (i.e., phonological awareness and decoding). For example, Tighe and Binder (2015) found that after controlling for phonological awareness, morphological awareness accounted for 33% unique variance in the reading comprehension skills of ABE students. Similarly, To et al. (in press) determined that morphological awareness contributed additional variance (7%) to reading comprehension beyond decoding skills in this population.

In addition, all of these studies assumed that morphological awareness represented a unidimensional construct as evidenced by the inclusion of a composite score of morphological awareness measures (Tighe and Binder, 2015; Fracasso et al., in press; To et al., in press). However, Tighe and Schatschneider (2015) investigated the dimensionality of morphological awareness in a sample of ABE students and reported that measures that included only pseudowords versus measures that included only real words represented distinct latent morphological factors. The real word morphological awareness and pseudoword morphological awareness factors were also separate from a latent vocabulary knowledge factor in this population. Moreover, real word morphological awareness exhibited a significantly stronger relationship with vocabulary knowledge (*r* = 0.68) than the relationship between pseudoword morphological awareness and vocabulary knowledge (*r* = 0.50). These findings suggest that the construct of morphological awareness is multidimensional and is separable from vocabulary knowledge in ABE students. Yet, none of the past research has investigated the shared and unique contributions of real word morphological awareness, pseudoword morphological awareness, and vocabulary knowledge to reading comprehension. Therefore, the current study extended previous research by: (1) administering multiple assessments and utilizing a latent variable modeling approach to account for measurement error; and (2) competing a model that treated morphological awareness as unidimensional against a model that treated morphological awareness as distinct dimensions of real word morphological awareness and pseudoword morphological awareness in predicting ABE students’ reading comprehension skills.

### Vocabulary Knowledge and Reading Comprehension

Vocabulary knowledge has also emerged as an important contributor to the reading comprehension skills of ABE students across several recent studies (Mellard et al., 2010; Mellard and Fall, 2012; Taylor et al., 2012; Hall et al., 2014; Fracasso et al., in press). Parallel to past research on morphological awareness, none of the vocabulary studies have employed a latent variable modeling approach to model measurement error and to explore a predictive model of reading comprehension. Further, the majority of these studies have included a single measure of vocabulary knowledge. For example, Mellard et al. (2010) and Hall et al. (2014) included only an expressive vocabulary measure; whereas Fracasso et al. (in press) included only a receptive vocabulary measure to predict adults’ reading comprehension skills.

Two studies on ABE students (Mellard and Fall, 2012; Taylor et al., 2012) have examined the predictive utility of a broader vocabulary construct (including expressive and receptive vocabulary measures) to predict reading comprehension. Mellard and Fall (2012) utilized a composite score of expressive and receptive vocabulary to predict individual differences in the reading comprehension skills of adults at three functional reading levels (beginning, intermediate, and secondary). This composite vocabulary score was not predictive for the beginning adult readers; however, accounted for roughly 25% of the variance in the intermediate adults’ reading comprehension skills and 50% of the variance in the secondary adults’ reading comprehension skills. Taylor et al. (2012) reported that oral vocabulary (comprised of expressive and receptive vocabulary measures) contributed 12% unique variance to reading comprehension after controlling for fluency and decoding skills. The current study extended previous findings by using a latent variable modeling approach with multiple measures of vocabulary knowledge (both expressive and receptive) to predict adults’ reading comprehension skills.

### Current Study

The purpose of the current study was twofold. First, the study investigated if morphological awareness is best represented as a unidimensional or two-dimensional construct in the prediction of the reading comprehension abilities of ABE students. To accomplish this, we compared a two-factor model (comprised of unidimensional morphological awareness and vocabulary knowledge factors) to a three-factor model (comprised of real word morphological awareness and pseudoword morphological awareness dimensions and a vocabulary knowledge factor). Building off of the findings of Tighe and Schatschneider (2015), we hypothesized that the three factors of real word morphological awareness, pseudoword morphological awareness, and vocabulary knowledge would emerge as important predictors of reading comprehension.

Second, the study explored the feasibility of fitting SEM models of reading comprehension to a sample of ABE students. To evaluate model effectiveness, we examined overall model fit indices as well as the joint and unique contributions of our morphological awareness and vocabulary factors to reading comprehension. Multiple studies have reported the importance of morphological awareness and vocabulary knowledge to ABE students’ reading comprehension skills (Taylor et al., 2012; Hall et al., 2014; Tighe and Binder, 2015; Fracasso et al., in press; To et al., in press); however, these constructs have not been represented together in a latent variable modeling framework. Thus, we hypothesized that the models would provide a good fit to our data and that the morphological and vocabulary factors would account for substantial variance in the reading comprehension skills of this population. Two primary research questions were addressed:

(1) How well does a two-factor model versus a three-factor model account for individual differences in the reading comprehension skills of ABE students?
(2) What are the magnitudes of the joint and unique estimates of the morphological awareness and vocabulary knowledge latent constructs to reading comprehension?

## Materials and Methods

### Participants

The participants included 136 native-English speaking adults enrolled in literacy classes at two centers in Northern Florida. This study was conducted in accordance with the rules of the Florida State University Institutional Review Board for Research Involving Human Subjects. All participants signed an informed consent form and we assigned participants a random identification number for data entry. The sample consisted of 51% females (*n* = 70) and a range of ages (16–73; *M* = 24). In addition, the participants represented a multitude of racial and ethnic backgrounds: 67.6% African American, 23.5% Caucasian, 5.1% Hispanic, 2.9% Mixed race, and 0.7% Asian. The educational background of the participants was diverse: 0.7% completed below a middle school level, 8.9% completed some middle school, 73.5% completed some high school, and 16.9% completed high school. The reading grade equivalencies (RGEs) of the sample ranged from 3 to 12.9 (*M* = 7.7; *SD* = 2.8) as assessed by the Reading subtest of the Test of Adult Basic Education (TABE). The RGEs were normally distributed across levels 2–6 of the National Reporting System (NRS): 8.1% at Level 2 (RGEs 2.0–3.9), 22.1% at Level 3 (RGEs 4.0–5.9), 36.8% at Level 4 (RGEs 6.0–8.9), 15.4% at Level 5 (RGEs 9.0–10.9), and 16.2% at Level 6 (RGEs 11–12.9). The skewness (0.12) and kurtosis (-0.72) values fell within an acceptable range.

### Measures

Participants were administered a battery of 10 tasks: seven experimental morphological awareness tasks, two norm-referenced vocabulary tasks, and a norm-referenced reading comprehension measure. Participants’ most recent TABE-Reading subtest scores were obtained from the adult literacy centers. Additionally, a demographic survey addressing age, employment status, and educational background was given.

#### Morphological Awareness

##### Base form morphology (BMORPH) task

This task assessed morphological structure by having participants decompose morphologically complex target words in order to identify the base morpheme. The examiner read aloud a target word, which served as a prime for the participant. Next, the examiner read aloud a sentence with a blank in it. The participant was asked to fill in the blank with the correct base word of the target word given. For example, “Election. Which person did they _____?”; “Elect.” Items were presented aloud and the participant had the written version of the task in front of them to avoid decoding, listening comprehension, and working memory difficulties. A correct response received one point and an incorrect response or no answer received zero points. Participants were given two practice items and 28 test items. The Cronbach’s alpha coefficient for the BMORPH was 0.86 for the sample.

##### Derived form morphology (DMORPH) task

This task was similar in layout to BMORPH and assessed morphological structure by having participants transform root words into morphologically complex derived words. The examiner provided participants with a root word followed by a sentence, which contained a blank. The participant was asked to fill in the blank with the appropriate complex derived word form of the root word supplied by the examiner. For example, “Explain. His excuse was a bad _____.”; “explanation.” Again, the participant had the task available to them visually while listening to the examiner. A correct response received one point and an incorrect response or no answer received zero points. Participants were provided with two practice items followed by 28 test items. The Cronbach’s alpha coefficient was 0.90 for the sample.

##### Derivational suffix choice test of pseudowords

This task assessed the ability to recognize appropriate derivational suffixes using pseudowords. A sentence with a blank was presented and the participant was prompted to select the correct answer from a list of four choices. For example, “He has too much _____.” The answer choices included “brinable,” “brinicity,” “brinify,” and “brinicious.” The correct answer “brinicity,” received one point while an incorrect answer or no answer received zero points. Items were read aloud to the participant and the participant had access to the written form. The task included one practice item followed by 18 test items. The Cronbach’s alpha coefficient was 0.85 for the sample.

##### Morphological skill task

This task assessed the ability to recognize morphological relatedness between derived and root words. The participant was presented with morphologically complex derived words followed by three root word answer choices. For example, a participant was provided with the complex word “readmission” and the three answer choices: “read,” “admit,” and “mission.” A correct response of “admit” elicited one point while an incorrect or no answer resulted in zero points. This task consisted of a two practice items followed by 29 test items. The Cronbach’s alpha coefficient was 0.75 for the sample.

##### Morphological construction task

This task measured the ability to manipulate syntactic information to construct new words. The participant was presented with mini scenarios, which contained a simple pseudoword and a blank. Based on the context of the scenario and the simple pseudoword, the participant was required to utilize inflectional knowledge to complete the blank with the appropriate pseudoword. For example, “This is a type of bird called a gutch. Now we have three of them. We have three _____.”; “gutches.” Correct answers received one point, incorrect or no answers received zero points. The task consisted of two practice items followed by 12 test items. The Cronbach’s alpha coefficient was 0.79 for the sample.

##### Morphological analogy real word task

This task followed the format of A : B :: C : D, in which the participant was presented with a pair of inflected words (either regular or irregular) and the first word of the second pair, “C.” The participant was asked to provide “D,” the second word from the second pair. An example was: “sit : sitting :: frame : _____.”; “framing.” A correct answer was given one point and an incorrect or no answer resulted in zero points. The task consisted of a practice round and 15 test items. The Cronbach’s alpha coefficient was 0.81 for the sample.

##### Morphological analogy pseudoword task

This researcher-created task followed the same A : B :: C : D as the morphological analogy task; however, the task included pseudowords. The participant was presented with a pair of real words and was then supplied with a pseudoword and asked to fill in the corresponding pseudoword. For example, “fuzz : fuzzy :: squilt : _____.”; “squilty.” A correct answer received one point and an incorrect or no answer received zero points. This task had a practice round followed by 15 items. The Cronbach’s alpha was 0.88 for the sample.

#### Vocabulary Knowledge

##### Peabody picture vocabulary test – fourth edition (PPVT-4)

The PPVT-4 is a norm-referenced measure of receptive vocabulary knowledge. The examiner presented a word aloud and the participant was asked to select the correct picture from four choices that best matched the meaning of the presented word. Testing commenced on set 11, item number 121. If greater than one error occurred in this initial set, testing continued with an easier set until a basal set was established. Once a basal set was obtained, testing continued (with sets increasing in difficulty) until eight errors were reached. The test was normed on individuals aged 3–90 and has a reported split-half reliability of 0.94 (Dunn and Dunn, 2007).

##### Expressive one-word picture vocabulary test – fourth edition (EOWPVT-4)

The EOWPVT-4 is a norm-referenced assessment of expressive vocabulary. The participant was presented with sets of pictures depicting objects, actions, and concepts. The participant was asked to provide a single-word name for each picture. Testing commenced on item number 85. A basal level was established once a participant correctly identified eight consecutive items and testing continued until six consecutive errors were made (ceiling level). This test was normed on individuals aged 2–103 years of age and has a reported median internal consistency reliability of 0.95 (Martin and Brownell, 2011).

#### Reading Comprehension

##### Test of silent reading efficiency and comprehension (TOSREC)

The TOSREC is a timed, norm-referenced measure designed to assess silent reading comprehension of connected text. The participant was presented with a series of sentences and was asked to indicate “yes” or “no” as to the truthfulness of the sentences. The participant was allotted 3 min to read silently and respond to as many sentences as possible. The test was normed on individuals in Grades 1–12 and has an alternate forms reliability 0.88 for the Grade 9 version (Wagner et al., 2010).

##### Test of adult basic education – reading (TABE)

The TABE is a widely used measure in adult literacy programs, which covers reading, math, language, mechanics, vocabulary, and spelling. The TABE consists of five levels: L (literacy, RGE = 0–1.9), E (easy, RGE = 1.6–3.9) M (medium, RGE = 3.6–6.9), D (difficult, RGE = 6.6–8.9), and A (advanced, RGE = 8.6–12.9). The Reading subtest requires adults to read brief passages and answer multiple-choice questions. The subtest includes narrative and expository texts as well as functional tests (i.e., reading a newspaper). The questions increase in difficulty at each level. For example, in the level L, the lowest level, participants are asked questions pertaining to recognizing letters and sounds, simple vocabulary words, and matching letters. Harder levels require participants to interpret graphic information, recall information, construct meaning, and generate inferences. The reading subtest contains 50 items and the internal consistency reliability ranges from 0.88 to 0.95 across the five levels (CTB/McGraw-Hill, 2008).

### Procedure

The 10 tasks were administered individually to the participants in two 30-min sessions over a 2-day span during Spring, 2012. Session one included the BMORPH, Suffix Choice, Analogy Real Word, PPVT-4, and TOSREC tasks. Session two included the DMORPH, Morphological Construction, Morphological Skill, Analogy Pseudoword, and EOWPVT-4 tasks. The order of the sessions and the order of the tasks within sessions were counterbalanced. By presenting the tasks over 2-days, we were able to eliminate time sampling error. Testing took place in a quiet classroom at each adult literacy center. Of the 136 participants, 127 completed both days of testing.

## Results

### Checking for Data Issues and Descriptive Statistics

Data was inspected for outliers, skewness, kurtosis, and missing values. Twenty univariate outliers across the 11 measures were identified and adjusted (brought to the boundary of the median +/- two interquartile ranges). An examination of scatterplots of all pairs of variables revealed no bivariate outliers. Skewness and kurtosis values fell within an acceptable range (±2), with the exception of BMORPH. A histogram indicated that BMORPH was leptokurtic, with a kurtosis value of 4.29 and a slight negative skew of -1.96. The BMORPH variable was transformed by reflecting it, taking the log transformation, and then reflecting it back. This transformation resulted in a kurtosis value of -0.45 and a skewness value of -0.53. Because there were relatively few missing data points (47 across the 11 measures) and no more than 7 in any single variable, maximum likelihood (ML) estimation was utilized.

Means, standard deviations, and ranges of the measures are reported in **Table 1**. **Table 2** presents the correlations between all of the measures. All measures were significantly and positively correlated (*p*s < 0.01).

**Table 1:** Descriptive statistics for all measures.

Measure	*N*	*M*	*SD*	Min/Max
**Morphological awareness**				
DMORPH	129	18.60	6.14	1–28
BMORPH	134	-0.64	0.25	-0.3 to -1.34
Morphological skill	129	21.74	4.08	11–28
Derivational suffix choice	134	11.73	4.53	1–18
Analogy real word	134	6.85	3.61	0–15
Analogy pseudoword	129	8.19	4.30	0–15
Morphological construction	129	9.25	2.49	4–12
**Vocabulary knowledge**				
PPVT-4	134	81.15	12.74	48–117
EOWPVT-4	129	72.81	14.38	55–111
**Reading comprehension**				
TOSREC	134	88.51	16.61	55–120
TABE-reading	134	541.92	53.20	422–676

*DMORPH, Derived Form Morphology; BMORPH, Base Form Morphology; PPVT-4, Peabody Picture Vocabulary Test- Fourth Edition; EOWPVT-4, Expressive One-Word Picture Vocabulary Test – Fourth Edition; TOSREC, Test of Silent Reading Efficiency and Comprehension; TABE, Test of Adult Basic Education.*

**Table 2:** Correlations among the measures.

Measure	1	2	3	4	5	6	7	8	9	10	11
(1) DMORPH	–	0.63	0.61	0.63	0.52	0.53	0.61	0.52	0.55	0.59	0.52
(2) BMORPH	–	–	0.55	0.65	0.56	0.47	0.48	0.44	0.47	0.57	0.43
(3) MSkill	–	–	–	0.55	0.49	0.50	0.43	0.36	0.45	0.54	0.53
(4) Suffix	–	–	–	–	0.56	0.53	0.63	0.33	0.37	0.54	0.47
(5) ARW	–	–	–	–	–	0.48	0.46	0.37	0.37	0.43	0.36
(6) APW	–	–	–	–	–	–	0.59	0.37	0.32	0.47	0.44
(7) Construct	–	–	–	–	–	–	–	0.36	0.32	0.53	0.51
(8) EOWPVT-4	–	–	–	–	–	–	–	–	0.83	0.53	0.40
(9) PPVT-4	–	–	–	–	–	–	–	–	–	0.59	0.48
(10) TOSREC	–	–	–	–	–	–	–	–	–	–	0.61
(11) TABE	–	–	–	–	–	–	–	–	–	–	–

*N = 125. BMORPH, Base Form Morphology; DMORPH, Derived Form Morphology; MSkill, Morphological Skill Task; ARW, Analogy Real Word Task; APW, Analogy Pseudoword Task; Construct, Morphological Construction Task; EOWPVT-4, Expressive One-Word Picture Vocabulary Test – Fourth Edition; PPVT-4, Peabody Picture Vocabulary Test- Fourth Edition; TOSREC, Test of Silent Reading Efficiency and Comprehension; TABE, Test of Adult Basic Education. All are significant at p < 0.01.*

### Structural Equation Models of Reading Comprehension

To address our research questions, we competed a two-factor model against a three-factor model and assessed the joint and unique contributions of our latent predictor variables to reading comprehension. All structural equation models were fit utilizing Version 7.31 of Mplus statistical software (Muthén and Muthén, 1998-2012). For identification purposes, our reading comprehension factor included two reliable observed indicators, TABE-Reading and TOSREC. Scale dependency was handled by fixing one indicator per latent variable to 1.0 (Kline, 2011). Finally, we relied on Hu and Bentler’s (1998) standards to determine good model fit: Tucker Lewis Index (TLI) and Comparative Fit Index (CFI) values greater than 0.95, Root Mean Square Error of Approximation (RMSEA) values less than 0.08, and Standardized Root Mean Square Residual (SRMR) values of less than 0.05.

**FIGURE 1:**
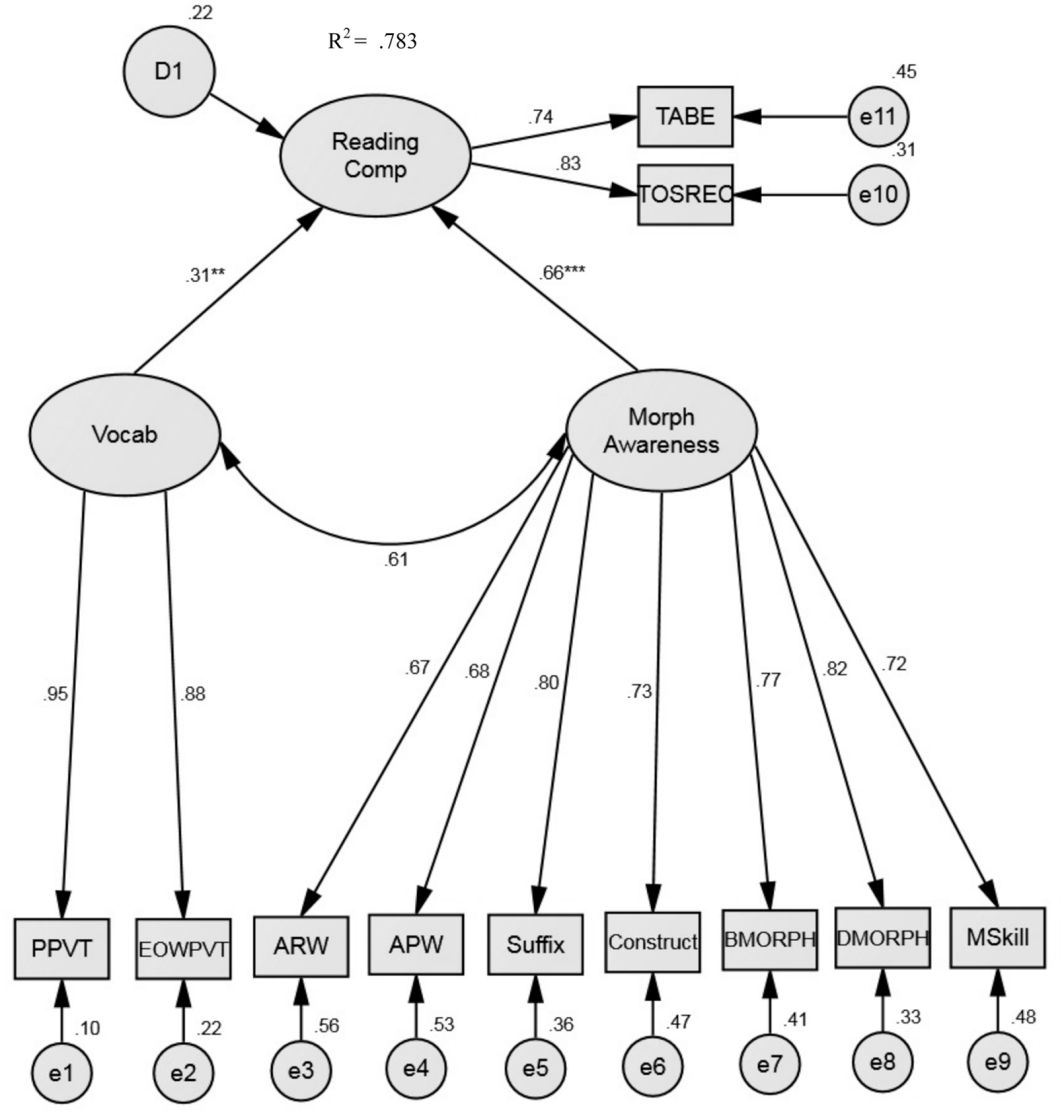
**Two-factor model of reading comprehension.** This model presents standardized parameter estimates. All loadings are significant at *p* < 0.001. TABE, Test of Adult Basic Education; TOSREC, Test of Silent Reading Efficiency and Comprehension; PPVT, Peabody Picture Vocabulary Test; EOWPVT, Expressive One-Word Picture Vocabulary Test; ARW, Analogy Real Word Task; APW, Analogy Pseudoword Task; Construct, Morphological Construction Task; BMORPH, Base Form Morphology; DMORPH, Derived Form Morphology; MSkill, Morphological Skill Task.

For our two-factor model, morphological awareness and vocabulary knowledge served as predictors of reading comprehension. Morphological awareness was conceptualized as a unidimensional factor, in which all seven morphological awareness tasks were observed indicators. The vocabulary knowledge factor included the two norm-referenced vocabulary assessments as indicators (**Figure 1**). This model provided adequate fit to the data, as evidenced by the model fit indices [χ^2^(41) = 68.51, *p* = 0.005, *CFI* = 0.967, *TLI* = 0.955, *RMSEA* = 0.070, and *SRMR* = 0.042; **Table 3**]. All factor loadings were significant (*p*s < 0.001) and all were above 0.66. The predictors of morphological awareness and vocabulary knowledge were moderately correlated (*r* = 0.61) and jointly accounted for approximately 78% of the variance of reading comprehension. Looking at **Figure 1**, it is apparent that when included in the same model, both morphological awareness and vocabulary knowledge contribute uniquely to predicting reading comprehension (β = 0.660, *p* < 0.001; β = 0.311, *p* = 0.001, respectively). In this model, morphological awareness accounts for roughly 29% unique variance and vocabulary knowledge accounts for roughly 5% of the unique variance in reading comprehension.

**Table 3:** Model fit indices for the models of reading comprehension.

Model	χ^2^(df)	*p*	CFI	TLI	RMSEA	SRMR
(1) Two-factor model	68.51(41)	0.005	0.967	0.955	0.070	0.042
(2) Three-factor model	53.96(38)	0.045	0.981	0.972	0.056	0.037

*χ^2^, chi-square statistic; df, degrees of freedom; CFI, Comparative Fit Index; TLI, Tucker-Lewis Index; RMSEA, Root Mean Square Error of Approximation; SRMR, Standardized Root Mean Square Residual*.

**FIGURE 2:**
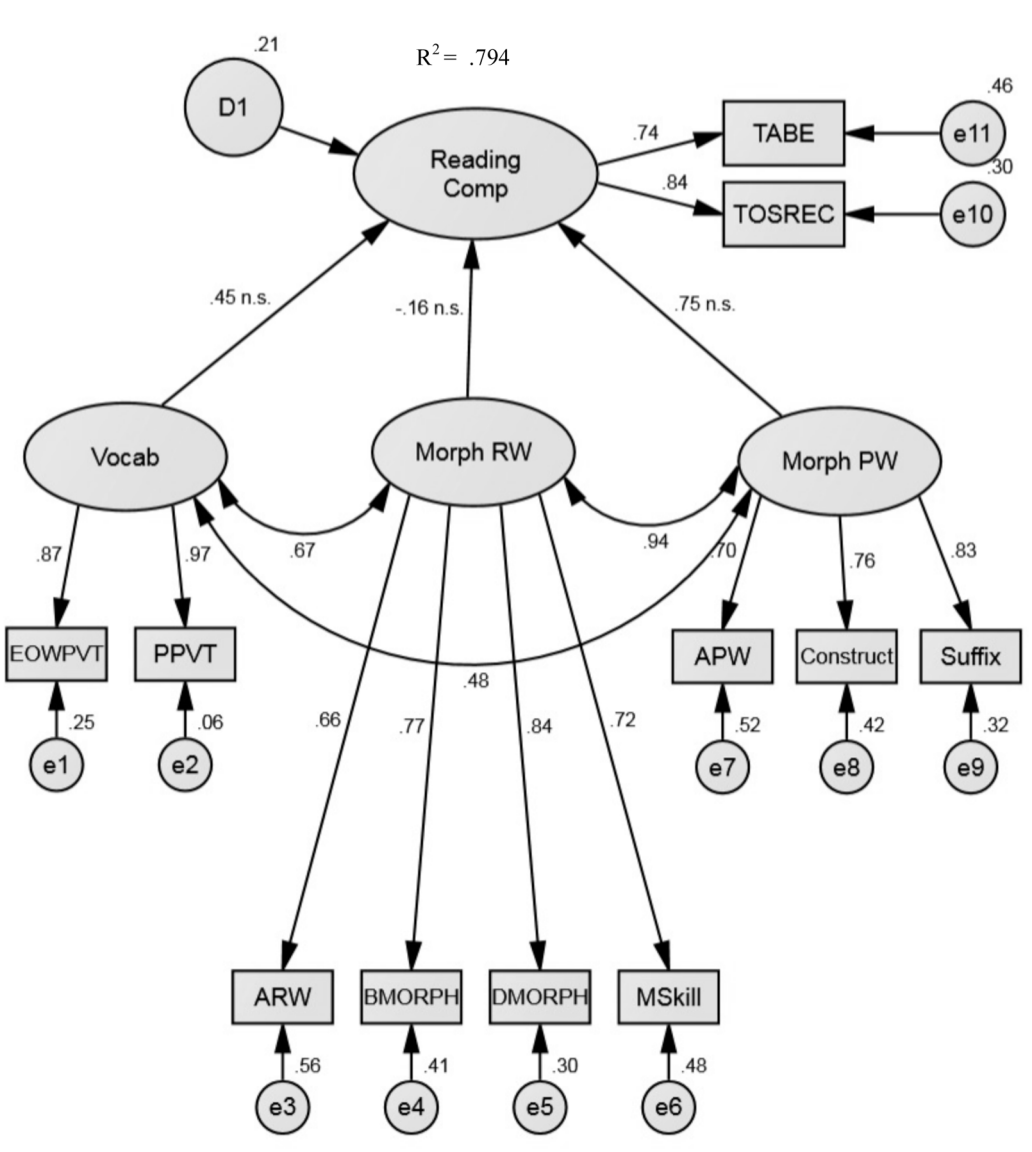
**Three-factor model of reading comprehension.** This model presents standardized parameter estimates. All loadings are significant at *p* < 0.001 unless otherwise specified (n.s., not significant.). TABE, Test of Adult Basic Education; TOSREC, Test of Silent Reading Efficiency and Comprehension; PPVT, Peabody Picture Vocabulary Test; EOWPVT, Expressive One-Word Picture Vocabulary Test; ARW, Analogy Real Word Task; BMORPH, Base Form Morphology; DMORPH, Derived Form Morphology; MSkill, Morphological Skill Task; APW, Analogy Pseudoword Task; Construct, Morphological Construction Task; Morph RW, Morphological Awareness Real Words; Morph PW, Morphological Awareness Pseudowords.

This two-factor model was compared to a three-factor model, which split morphological awareness into separate dimensions of real word and pseudoword morphological awareness and retained the vocabulary knowledge factor. Our three-factor model was formulated based on previous research (Tighe and Schatschneider, 2015), which reported that morphological awareness represents a two-dimensional construct in this population. For the three-factor model, the real word morphological awareness factor contained four indicators (Analogy Real Word, BMORPH, DMORPH, and Morphological Skill) and the pseudoword morphological awareness factor contained three indicators (Analogy Pseudoword, Suffix Choice, and Morphological Construction). Again, the vocabulary knowledge factor had two indicators (EOWPVT-4 and PPVT-4; **Figure 2**). This model provided good fit to the data as observed by the model fit indices [χ^2^(38) = 53.96, *p* = 0.045, *CFI* = 0.981, *TLI* = 0.972, *RMSEA* = 0.056, and *SRMR* = 0.037; **Table 3**). Additionally, all factor loadings were significant (*p*s < 0.001] and were all above 0.65. A chi-square difference test revealed that the three-factor model provided significantly better fit to the data [χ^2^(3) = 14.55, *p* = 0.002] than the two-factor model. This replicates the finding from Tighe and Schatschneider (2015) that morphological awareness remains a two-dimensional construct in the presence of vocabulary knowledge and extends the findings by including reading comprehension in the model.

Jointly, the latent predictor variables (real word morphological awareness, pseudoword morphological awareness, and vocabulary) of the three-factor model accounted for approximately 79% of the variance in reading comprehension. In **Figure 2**, it appears as though real word morphological awareness and pseudoword morphological awareness are not significant predictors of reading comprehension (β = -0.161, *p* = 0.846; β = 0.750, *p* = 0.284, respectively) and that vocabulary is a marginally significant predictor of reading comprehension (β = 0.451, *p* = 0.052). In conjunction with all other predictors in the model, real word morphological awareness and pseudoword morphological awareness do not account for additional (or unique) variance in reading comprehension. Vocabulary is marginally significant and may contribute uniquely (5.6%) to reading comprehension beyond real word and pseudoword morphological awareness. Possibly as a result of the high collinearity among the dimensions of morphological awareness, none of the factors accounted for unique significant variance in reading comprehension. However, in a series of models where each predictor of reading comprehension was included separately, the factors all account for independent, significant variance in reading comprehension: real word morphological awareness (β = 0.867, *p* < 0.001, *R*^2^ = 0.752), pseudoword morphological awareness (β = 0.833, *p* < 0.001, *R*^2^ = 0.695), and vocabulary (β = 0.695, *p* < 0.001, *R*^2^ = 0.483). It is also worth noting that the real word morphological awareness and reading comprehension factors were as highly correlated (*r* = 0.84) as the pseudoword morphological awareness and reading comprehension factors (*r* = 0.82). Thus, we conclude that real word morphological awareness and pseudoword morphological awareness are necessary facets of the broader construct of morphological awareness and that both dimensions are important to reading comprehension. Moreover, it is important to differentiate between dimensions of morphological awareness and vocabulary knowledge in a model of reading comprehension for ABE students.

## Discussion

The first aim of the current study was to follow-up on the dimensions of morphological awareness proposed in Tighe and Schatschneider (2015) by comparing a model that treated morphological awareness as unidimensional to a model that treated morphological awareness as two-dimensional (separate real word morphological awareness and pseudoword morphological awareness factors) in predicting reading comprehension. The second aim of the study was to examine the magnitudes of the shared and unique contributions of the latent predictor variables to the reading comprehension skills of ABE students. Our results indicated that a three-factor model with real word morphological awareness, pseudoword morphological awareness, and vocabulary knowledge factors provided the most parsimonious fit to our sample. Moreover, these factors jointly accounted for a substantial portion (79.4%) of the variance in reading comprehension. Vocabulary knowledge emerged as the only potentially unique predictor (*p* = 0.052); however, isolating the factors of real word morphological awareness and pseudoword morphological awareness indicated that these were significant individual predictors of reading comprehension. These findings provide evidence for the importance of morphological awareness (real and pseudoword) and vocabulary knowledge to reading comprehension in this population.

### Two-Factor versus Three-Factor Reading Comprehension Models

Past research has consistently identified morphological awareness (Tighe and Binder, 2015; Fracasso et al., in press; To et al., in press) and vocabulary knowledge (Mellard et al., 2010; Mellard and Fall, 2012; Taylor et al., 2012; Hall et al., 2014) as important predictors of the reading comprehension skills of ABE students. However, none of this research has utilized a latent variable modeling approach in order to: (a) estimate the underlying nature of these constructs and take into account measurement error; and (b) predict individual differences in reading comprehension skills using multiple measures. Further, all of these studies have treated morphological awareness as a unidimensional construct in predicting reading comprehension. Tighe and Schatschneider (2015) utilized confirmatory factor analyses (CFAs) to examine the dimensionality of morphological awareness and the relationship between morphological awareness and vocabulary knowledge in a sample of ABE students. Tighe and Schatschneider (2015) found that a three-factor CFA comprised of real word morphological awareness, pseudoword morphological awareness, and vocabulary knowledge factors provided the best fit to the data. This finding is in contrast to past research conducted with children in which morphological awareness was found to be a unidimensional construct (Muse, Unpublished) and not separable from vocabulary knowledge (Muse, Unpublished; Spencer et al., 2015). The current study extended the findings of Tighe and Schatschneider (2015) by including separate latent dimensions of morphological awareness as well as vocabulary knowledge in a structural equation model (SEM) of reading comprehension. We tested a two-factor (morphological awareness and vocabulary knowledge) model against a three-factor (real word morphological awareness, pseudoword morphological awareness, and vocabulary knowledge) model. In accordance with Tighe and Schatschneider (2015), the three-factor model that treated morphological awareness as two-dimensional emerged as the best fit to our data.

We applied SEM models as an alternative to a CFA approach to modeling the relations among component skills and investigating predictors of reading comprehension. Our three factors jointly accounted for 79.4% of the variance in reading comprehension in this population. Vocabulary was marginally non-significant (*p* = 0.052), but contributed an additional 5.6% unique variance to reading comprehension after controlling for real word morphological awareness and pseudoword morphological awareness. Our small sample size (*N* = 136) may have restricted our power to detect a significant effect. Real word morphological awareness and pseudoword morphological awareness did not account for additional unique variance possibly because of the high collinearity between the two dimensions of morphological awareness. However, this should not be taken as evidence that the dimensions of morphological awareness are unimportant to reading comprehension skills in this sample. In fact, isolating the real word and pseudoword morphological awareness factors revealed that separately each contributed significant variance to reading comprehension (75% and 70%, respectively). Thus, all three factors are essential to understanding a model of reading comprehension in ABE students.

### Implications of Findings for Educators and Researchers in ABE Programs

Our findings have important implications for educators and researchers in ABE contexts. For ABE educators, the findings add to the limited body of research on core reading component skills in this population. The three-factor model illustrates the importance of incorporating real word morphological awareness, pseudoword morphological awareness, and vocabulary knowledge into instructional practices. Past research with children across varying grade levels, has indicated that explicit morphological awareness instruction builds vocabulary knowledge (Bowers and Kirby, 2010; Goodwin and Ahn, 2010, 2013), word reading (Katz and Carlisle, 2009; Goodwin and Ahn, 2013) and reading comprehension skills (Katz and Carlisle, 2009; Bowers et al., 2010; Goodwin and Ahn, 2010). Thus, explicit morphological awareness instruction may promote growth in several component reading skills as well as reading comprehension skills and should be explored in ABE programs. Moreover, past research has identified that compared to achievement-matched children, ABE students compensate for weak phonological decoding skills (i.e., pseudoword decoding) by relying on contextual cues and other metalinguistic abilities (i.e., morphological and orthographic cues; Greenberg et al., 1997, 2002; Thompkins and Binder, 2003). Thus, morphological instruction (particularly in pseudoword morphological awareness) may aid adults exhibiting deficits in phonological processing skills.

For researchers, our findings demonstrate the feasibility of fitting SEM models of reading comprehension in an ABE sample. SEM models allowed us to examine the predictive relations of three constructs to reading comprehension and to assess the magnitudes of the unique and shared variances estimates. The three constructs accounted for a substantial proportion of the reading comprehension variance (79%), which contributes to our understanding of the underlying component reading skills in this understudied population. Moreover, these models provide insight into assessing the construct validity of morphological awareness. The results indicate that to fully assess the construct of morphological awareness researchers and practitioners need to include items and measures that assess real word and pseudoword morphological awareness. Further, the results provide preliminary evidence that morphological awareness (real and pseudoword morphological awareness) and vocabulary knowledge may be central constructs to target for interventions with this population.

### Limitations and Future Directions

Two limitations should be addressed. First, additional component reading skills should be considered to build the most comprehensive model of reading comprehension in this population. The three latent factors accounted for a large portion of the variance in reading comprehension; however, an additional 20% of the variance in reading comprehension remains unexplained. For example, past research with ABE students has investigated the contribution of morphological awareness controlling for phonological awareness (Tighe and Binder, 2015) and decoding skills (To et al., in press). In the current study, real word morphological awareness seems to overlap more with vocabulary knowledge (*r* = 0.67) than the overlap between pseudoword morphological awareness and vocabulary knowledge (*r* = 0.48). Thus, pseudoword morphological awareness may be more related to an individual’s phonological awareness or pseudoword decoding knowledge; whereas, real word morphological awareness may be more related to an individual’s vocabulary knowledge and real word decoding skills. Additionally, past research with children has identified different latent dimensions of vocabulary knowledge (see Tannenbaum et al., 2006; Kieffer and Lesaux, 2012). These nuances in vocabulary knowledge should be explored in an ABE sample and incorporated into future models of reading comprehension.

Second, the current study utilized a small sample of native English speaking adult literacy students. This sample is not representative of ABE programs in the U.S. because approximately 43% of these programs include non-native English speakers (Lesgold and Welch-Ross, 2012). It is not clear whether native and non-native English speakers enrolled in ABE programs have similar literacy profiles. Therefore, future research should investigate optimal predictors of reading comprehension for both native and non-native students. Further, it would be useful to include a larger sample size and examine the extent to which these findings generalize to different RGEs in ABE samples.

## Conclusion

The current study has begun to shed light on the importance of morphological awareness (real word and pseudoword morphological awareness) and vocabulary skills to reading comprehension in an under-studied population of adults with low literacy skills. Moreover, the study demonstrates the utility of fitting latent variable models of reading comprehension to an ABE sample. These findings have important implications for incorporating the explicit teaching of real word morphological awareness, pseudoword morphological awareness, and vocabulary knowledge in ABE programs. Future research should strive to understand the relations among additional component reading skills in this population in order to build a complete model of reading comprehension.

## Author Contributions

All authors listed, have made substantial, direct and intellectual contribution to the work, and approved it for publication.

## Conflict of Interest Statement

The authors declare that the research was conducted in the absence of any commercial or financial relationships that could be construed as a potential conflict of interest. The reviewer Jose Manuel Tomás and handling Editor declared their shared affiliation, and the handling Editor states that the process nevertheless met the standards of a fair and objective review.
